# Challenges and opportunities for oral pre-exposure prophylaxis in the prevention of HIV infection: where are we in Europe?

**DOI:** 10.1186/1741-7015-11-186

**Published:** 2013-08-23

**Authors:** Jean-Michel Molina, Claire Pintado, Caroline Gatey, Diane Ponscarme, Pierre Charbonneau, Benedicte Loze, Willy Rozenbaum, Constance Delaugerre

**Affiliations:** 1 INSERM U941, University of Paris Diderot, Sorbonne Paris Cité, Paris, France; 2 Laboratory of Virology, Hopital Saint Louis, AP-HP, Paris, France; 3 Department of Infectious Diseases, Hopital Saint Louis, AP-HP, 1 avenue Claude Vellefaux, Paris 75010, France

**Keywords:** HIV, Tenofovir, Emtricitabine, Men who have sex with men, Intermittent, PrEP, Adherence

## Abstract

Following US Food and Drugs Administration approval in July 2012 of daily oral tenofovir and emtricitabine for pre-exposure prophylaxis (PrEP) to prevent HIV infection in high-risk individuals in the USA, there has been much controversy about the implementation of this PrEP regimen in other countries throughout the world, and in Europe in particular. In this review, we focus on the challenges and opportunities of a daily oral PrEP regimen to curb the rising incidence of HIV infection in high-risk groups, and particularly in men who have sex with men. A number of issues would need to be addressed before PrEP could be implemented, including assessing the real effectiveness and cost-effectiveness of daily PrEP, the sustainability of daily adherence, the risk of selecting resistance, the long-term safety, and the risk of change in sexual behavior that might offset the benefit of PrEP. Alternatives to a daily oral PrEP regimen are being explored.

## Introduction

Pre-exposure prophylaxis (PrEP) is a new biomedical intervention to prevent HIV acquisition in HIV-seronegative high-risk individuals using anti-retroviral drugs before HIV exposure.

In the wake of the recent US Food and Drug Administration (FDA) approval of a combined pill of tenofovir disoproxil fumarate (TDF) and emtricitabine (FTC) for daily PrEP to prevent sexual acquisition of HIV in high-risk individuals, there has been much controversy both in the medical and in men who have sex with men (MSM) about implementation of PrEP [1]. In a recent online survey of readers of the *New England Journal of Medicine*, only 51% of 1,115 respondents from 85 countries voted for the initiation of PrEP in a 46-year old man who has sex with men, multiple sexual encounters, and who is asking whether he should receive PrEP [2]. Even in the USA, the uptake of PrEP has been lower than expected, owing in part to limited awareness, and a number of demonstration projects are being implemented to assess real-life acceptability and adherence to a daily PrEP regimen in high-risk individuals, mostly in men who have sex with men (MSM) [3]. In Europe, PrEP is not yet approved, and research is still ongoing to assess PrEP among MSM.

In this review, we focus on the various issues that would need to be addressed before oral daily PrEP could be implemented on a large scale and become a worldwide public-health strategy for HIV prevention, particularly among high-risk MSM and particularly in Europe.

### Is there a need for PrEP in Europe?

Although the number of new HIV infections is slowly decreasing in many European countries, there has been no decline and even a small increase of new HIV infections in MSM. In France, for example, MSM account for up to 40% of new HIV infections [4], and this is the only risk group in which the prevalence of HIV infection has increased over the past few years. Similar reports from the UK show a similar rising number of new HIV infections in MSM, despite an increasing number of these individuals being tested for HIV, and more HIV-infected patients receiving antiretroviral therapy (ART), which results in suppressed viral replication [5]. With an HIV incidence in MSM that is 200-fold higher than that in the general population and a concomitant increase in other sexually transmitted infections (syphilis, gonorrhea, chlamydia, and hepatitis C) there is a clear need for strengthening prevention in this high-risk group [6]. Although use of the currently available prevention tools (information and education, regular use of condoms, change in sexual behavior, regular testing for HIV, ART for the HIV-infected partner, post-exposure prophylaxis with ART started immediately after at-risk sexual intercourse) needs to be reinforced, new tools such as PrEP might be an opportunity to foster prevention in MSM in Europe, as no HIV vaccine is yet available and male circumcision has not been shown to prevent HIV transmission via the anal route.

### Do we have enough confidence in PrEP effectiveness?

To date six large phase III efficacy trials of oral PrEP with TDF or TDF/FTC have been conducted in high-risk individuals, but have yielded conflicting results [7-12] (Table 1). Indeed, whereas all trials had a similar placebo-controlled design and assessed the benefit of daily oral PrEP on HIV incidence, efficacy outcomes ranged from a 75% reduction of HIV incidence among serodiscordant couples in the Partners PrEP study, to a non-significant 49% increase in HIV incidence in the TDF arm of the VOICE (Vaginal and Oral Interventions to Control the Epidemic) trial in high-risk young women. Only a single trial, iPrEx (Chemoprophylaxis for HIV Prevention in Men who have Sex with Men), has been carried out in MSM [7]. In this trial, for which participants were mainly enrolled from low-income and middle-income countries in South America, the overall efficacy was a 44% reduction in HIV incidence, but the lower bound of the 95% confidence interval of treatment efficacy was only 15%, below the predefined efficacy target of 30% [7]. Indeed, 30% is usually considered by regulatory authorities to be the lowest level at which a preventive intervention would be associated with a public-health benefit [13]. Such inconsistency was not found in the three randomized trials assessing the benefit of male circumcision for HIV prevention in heterosexual men, where a similar 60% reduction of HIV incidence was found with this one-time intervention, which has now been implemented as a public-health strategy in a number of countries with high endemic rates of HIV to reduce incidence [14].

**Table 1:** Efficacy and adherence rates across PrEP trials

**Study [reference]: countries**	**Population**	***n***	**Efficacy**	**Lower bound of 95% CI**	**Adherence**^**a**^
Partners PrEP [8]: Kenya, Uganda	Heterosexual couples	4758	67% TDF; 75% TDF/FTC	44% TDF; 55% TDF/FTC	82%
TDF2 Study [9]: Botswana	Young men and women	1219	62% TDF/FTC	21.5%	80%
Bangkok TDF [12]: Thailand	IVDU	2413	49% TDF	9.6%	67%
iPrEx [7]: S. America, SA, Thailand, USA	MSM	2499	44% TDF/FTC	15%	51%
FEM-PrEP [10]: Kenya, SA, Tanzania	Young women	2120	6% TDF/FTC	−52%	37%
VOICE [11]: South Africa, Uganda, Zimbabwe	Young women	5029	−49% TDF; - 4% TDF/FTC	−130% TDF; −50% TDF/FTC	30%

Abbreviations: FTC, emtricitabine; IVDU, intravenous drug users; MSM, men who have sex with men; PrEP, pre-exposure prophylaxis; SA, South Africa; TDF, tenofovir disoproxil fumarate.

^**a**^Adherence was assessed by the proportion of participants with drugs detectable in plasma and who remained free of infection in the active PrEP arms.

These discrepant results for PrEP effectiveness have led European regulatory authorities to defer approval of oral PrEP pending the results of ongoing PrEP trials being conducted in Europe (PROUD, IPERGAY) and those of open-label clinical trials (IPrEX Open Label Extension (IPrEx-OLE) and Partners PrEP extension) and demonstration projects in the USA. Indeed, despite FDA approval, which was granted before the full results of the VOICE trial were available, there is currently a low uptake of PrEP in the USA. More evidence is therefore needed to show the real effectiveness of oral PrEP, particular among MSM, before implementation in Europe.

### Why have there been conflicting results across PrEP trials?

The reasons for these discrepant results between PrEP trials are not completely straightforward, and a number of explanations have been proposed.

Like any medical intervention, PrEP works only when it is taken, and we have learned from HIV-infected patients how adherence to ART is crucial for achieving optimal outcomes. The same is also true for PrEP, as its efficacy in trials seemed to be strongly correlated to adherence with this daily regimen (Table 1). Therefore, differences in adherence rates between PrEP trials are likely to be the main reason for these discrepant efficacy results. Indeed, adherence, as measured by the proportion of patients with drug levels detectable in plasma ranged from 82% in the Partners PrEP study to as low as 30% in VOICE. We also learned from these trials that adherence measured by self-report or pill count was not reliable, and overestimated real adherence as measured by plasma drug levels. In iPrEx, adherence measured by plasma drug level was only 51%, but *post hoc* analyses showed that only 7% of those infected in the active arm had the drugs detectable in plasma at the time of infection, which the authors translated into a 92% (95% CI 40–99) efficacy of PrEP in those with drugs detected in plasma [7]. However, such a *post hoc* analysis is no longer protected by randomization, and those individuals with high adherence to PrEP might also be those most adherent to the other preventive tools made available in the trial. Because it is not possible to compare HIV incidence among patients with high adherence to both PrEP and placebo (although including a tracer in the placebo could be an option), such an analysis should be taken with caution. Indeed, 31% of participants in the active arms of the Partners PrEP trials became infected while having detectable, sometimes high, levels of drugs in their plasma, and such a correlation between plasma drug levels and treatment efficacy did not seem to be present in the VOICE trial [8,11].

Other explanations for these conflicting results have also been proposed as there are major differences between these trials in terms of gender, age, route of HIV acquisition, and rate of concomitant sexually transmitted infections among participants.

Young MSM (<25 years) in IPrEx had a two-fold higher risk of HIV acquisition and also were more than three time less likely to be adherent to PrEP [15]. In addition, because the trials that failed (Fem-PrEP and VOICE) were carried out in young women in sub-Saharan Africa, it is therefore possible that this PrEP strategy may be less effective in women. Although no significant difference in terms of efficacy between men and women was reported in the Partners PrEP trials, there was a non-significant trend toward a lower efficacy of this strategy in women than men with TDF/FTC (66% versus 84%) but not with TDF alone (71% versus 63%) [8]. Nevertheless, should it be confirmed that there is a difference in PrEP efficacy between men and women, this could be explained by the route of HIV acquisition and the differential pharmacokinetics of these antiretroviral drugs in the vaginal and rectal tissues. Indeed, pharmacokinetics studies in healthy volunteers following oral dosing with TDF/FTC have shown a 20-fold to 100-fold higher exposure to TVF-DP (the phosphorylated active metabolite of TDF) in rectal tissue compared with blood or vaginal and cervical tissues [16].

Other factors associated with an increased risk of sexual transmission of HIV might also be relevant to explain the different outcomes of these various PrEP trials. In studies performed in sub-Saharan Africa, younger age, high plasma HIV viral load in the HIV-infected partner, lower use of condoms, and incidence of sexually transmitted infections (STIs), whether symptomatic or asymptomatic in the uninfected partners, were all independently associated with a higher risk of HIV transmission [17,18]. It is therefore possible that HIV-seronegative participants in the Partners PrEP trials, who were in a stable couple relationship for several months were less exposed to HIV-infected partners with primary HIV infection, which is a period of high risk for HIV transmission because of very high viral loads in plasma and genital secretions. In addition, the number of sexual partners, and therefore the prevalence of STIs, is likely to be much lower among stable couples than among young men and women. The same statement could apply to the use of condoms and sexual behavior in general, which might explain why PrEP may work better in a setting where the risk of HIV transmission per sexual act is lower.

### Is oral PrEP safe enough?

There is a considerable weight of data available on the safety of TDF/FTC as daily oral PrEP, and has so far been reassuring, although the follow-up period in these PrEP studies has been limited so far to a couple of years. This safety profile is not unexpected, as TDF and FTC have long been used for the treatment of HIV infection and are considered the drugs of choice not only because of their potent antiviral activity but also because of their long-term safety. In terms of initial tolerability, participants receiving PrEP have experienced more nausea and diarrhea compared with those receiving placebo. Overall, there were no more study treatment discontinuations in the active arms than in the placebo arms of PrEP trials [7-12].

However, renal and bone toxicities are the two long-term safety issues that need to be monitored in patients taking TDF. Indeed, in previous PrEP trials, a few participants had to discontinue treatment because of increased creatinine levels, which usually returned to normal once the drug was discontinued. In any case, only people with normal creatinine clearance should receive TDF, and both glomerular and tubular functions need to be monitored regularly during TDF treatment. Similarly, small reductions in bone-mineral density have been reported in healthy participants of PrEP trials receiving a TDF-containing PrEP regimen, but the clinical relevance of this currently remains unknown [19].

The major threat of PrEP use is the risk for selecting HIV drug-resistance-associated mutations. This selection of resistance is of particular concern because both TDF and FTC are the cornerstone of antiretroviral therapy today, and their efficacy would be greatly jeopardized by the emergence of such resistance mutations. Although many options are available today for the treatment of patients with HIV infection, even in cases of drug-resistant viruses, every effort should be made to avoid this risk of selecting resistance. So far, in clinical trials, this risk of selecting for HIV drug resistance among participants who became infected despite PrEP has been low, in the range of 7% of those assigned to receive PrEP [7-12]. In fact, the large majority of participants who developed resistance to TDF or FTC were those who were already infected at the time they started PrEP, and it was expected that receiving a dual combination of anti-retrovirals could lead to the emergence of resistance. That is the reason why it is of utmost importance to exclude HIV infection before starting any patient on PrEP, and we know that current serological assays, especially rapid tests, can miss primary HIV infection [20]. It is therefore essential to defer PrEP prescription in a person who has symptoms suggestive of primary HIV infection and to perform PCR assays to detect HIV RNA in blood.

### What is the cost-effectiveness of oral PrEP?

Few studies have addressed the crucial issue of cost-effectiveness with the use of a daily oral PrEP regimen of TDF and FTC. As a prerequisite for such cost-effectiveness analyses, the strategy obviously needs to be effective in trials. Here, we focus on cost-effectiveness studies in MSM, based on the IPrEx results.

The first study looked at the cost-effectiveness of daily PrEP for MSM in the USA using a dynamic model of HIV transmission and progression with a detailed economic analysis [21]. Benefits and costs of PrEP were then assessed over 20 years of PrEP use by MSM. If 20% of all MSM were to use PrEP, more than 62,000 new cases of HIV infection would be prevented, with a resulting declining prevalence of HIV by 10% at 20 years compared with no PrEP [21]. However, the incremental cost for the healthcare budget would be significant (USD 95 billion), with a cost of more than USD 172,000 per quality-adjusted life-year (QALY) much higher than would be considered to be a cost-effective strategy. However, if PrEP were to be used by 20% of those at high risk (defined as those with more than 5 partners per year), 41,000 cases of HIV would be prevented, with a similar reduction of HIV prevalence by 10% at 20 years. This strategy would be a cost-effective intervention, because it would cost approximately USD 40,000 per QALY gained; however, it would still be associated with an increase in healthcare expenditure of about USD 14 billion over 20 years. It should be noted that the authors warned that their sensitivity analysis indicated that if there were to be a decrease of 20% in condom use, a paradoxical increase of 4% in new HIV infections could occur.

Another assessed the cost-effectiveness of this strategy in Peru, where most recruitment in the IPrEx study took place. In that study, the daily PrEP strategy with TDF/FTC would not be cost-effective using the World Bank threshold at the current cost of TDF/FTC [22]. Only certain optimistic scenarios combining a low coverage of only 5% of MSM with high prioritization to those at higher risk could be cost-effective.

These data help to explain the current reluctance of health authorities in a number of countries to implement PrEP, and this also applies to Europe. Furthermore, the issue of reimbursement is a sensitive one, as the principles of access equity should apply to newly approved drugs.

### What is the risk associated with risk compensation during PrEP use?

Risk compensation, which in this case can be defined as a sexual behavior with higher risk for HIV acquisition (for example, reduced condom use or condomless sex, increasing number of sexual partners), is a possible factor that could jeopardize current efforts in the field of HIV prevention. Theoretically, people using PrEP might feel protected against HIV and therefore be less prone to use condoms, or be willing to extend the number of their sexual partners. Online surveys among MSM indicate that this could be indeed the case. In a French study, up to 27% of respondents reported that they might stop condom use and 42% that they might reduce condom use if PrEP were to become available [23]. In addition, some respondents feared that the availability of PrEP might encourage their sexual partners to ask for condomless sex. Hence, there is a general concern that PrEP availability might decrease condom use, which could therefore offset the potential benefit of this therapy in preventing HIV infection, and ultimately this could even lead to an increase in the number of new HIV infections.

However, it should be noted that none of the PrEP trials to date found evidence of sexual disinhibition, and this finding was reasonably consistent across trials [7-11]. In fact, there was, on the contrary, a small but significant decrease in the number of receptive anal intercourse and a small but significant increase in condom use during the course of the IPrEx trial compared with baseline [7].

This reduction in high-risk sexual behavior in all PrEP trials is likely to be a consequence of the close counseling that participants involved in those trials received. It would therefore be essential to provide this same kind of support outside trials to avoid the risk of disinhibition. It should also be remembered that in all the placebo-controlled PrEP trials to date, the participants did not know whether they were receiving an active drug or a placebo, and therefore might have been more receptive to counseling. Whether this would hold true in real-life settings needs further study and the open-label extension phases of the IPrEx and Partners PrEP trials should be informative in that respect.

### Is the high level of adherence required with daily PrEP sustainable?

If we assume that the efficacy of PrEP is associated with high adherence rates, then based on previous studies, treatment should aim at an adherence rate of at least 80%, as observed in Partners PrEP [8].

It is interesting to analyze the reasons why adherence was so high in that trial compared with others performed in similar settings in sub-Saharan Africa. After conducting in-depth qualitative interviews, Ware *et al*. elegantly identified a number of factors that might explain the differences in adherence rates between a seronegative partner in an HIV-serodiscordant couple, and an unmarried man or woman [24]. Within a serodiscordant couple, there is a ‘discordant dilemma’ for the seronegative partner: trying to avoid HIV-infection while preserving the relationship in a context of desire for children and inconvenient long-term use of condoms. In these couples, PrEP can be seen as a solution, safeguarding health without ending the relationship. PrEP users are also likely to benefit from the support of their HIV-infected partners to improve their adherence. This is in sharp contrast with studies of young MSM, whose adherence to daily PrEP waned over a period of only 6 months from 63% to only 20% [25].

There is also demographic and geographic variability in adherence across PrEP trials, with older participants and those enrolled in sites in the USA showing usually higher adherence rates compared with younger participants or those enrolled in non-USA sites [7]. Eventually, it will be essential to assess PrEP adherence in open-labeled extensions of placebo-controlled trials or of demonstration projects. Indeed, adherence might be higher among people willing to take oral PrEP who are aware of the benefit shown in PrEP trials if participants had high rates of adherence to a daily regimen. In this regard, recent data from participants of the open label extension of the iPrEx study (IPrEx-OLE), showing an increase in adherence rate (measured by drug detection in plasma) from 61% during the placebo-controlled phase of the trial to 71% in the open-label extension, is reassuring [26].

Therefore ways to improve adherence to PrEP are needed if this strategy is to be successful. Providing long-term support for adherence will be crucial even if adherence might be higher in real life than it was in trials as a result of the known efficacy of PrEP. Monitoring adherence during PrEP will also be key, although data on adherence assessed by self-report or pill count are not fully reliable. Real-time monitoring of plasma drug levels could be an option, and new and more reliable assays based on measurements of tenofovir-diphosphate (TFV-DP) or FTC-triphosphate (FTC-TP) in peripheral blood mononuclear cells (PBMCs) or red cells using dry blood spots are being developed, which are likely be useful in developing countries in particular [27]. Assays have also been devised to measure drug exposure in hair [28].

### Alternatives to daily oral PrEP

Following the first encouraging results of PrEP trials, and FDA approval of TDF/FTC for PrEP, research in this area has been exploding. New oral drugs are being tested, as well as new drug combinations. Maraviroc, an HIV entry inhibitor already approved for the treatment of HIV infection, has entered clinical trials. Maraviroc can be dosed once daily, has a good safety profile, and achieves high levels in vaginal secretions and rectal tissue. Its safety and pharmacokinetics is currently being assessed in the HPTN 069 trial (Next-PrEP), both alone and in combination with TDF or FTC, in high-risk MSM and women.

As more acceptable PrEP regimens are being developed to improve adherence, there has been great interest in intermittent PrEP. Indeed, in animal models, oral intermittent PrEP, given at the time of virus inoculation, whether by vaginal or rectal challenge, provided an efficacy similar to that provided by use of daily PrEP [29]. This strategy of coitus-dependent PrEP is currently being assessed in two PrEP trials in MSM, under the assumption that the convenience of the regimen could increase PrEP adherence and therefore PrEP efficacy [30,31]. Interestingly, in young heterosexual women, coitus-dependent use of TDF gel was able to significantly reduce the incidence of HIV infection, whereas daily use of TDF gel in a similar population failed to show a significant benefit, suggesting that the convenience of the regimen plays an important role in PrEP adherence [11,32]. Should it prove to be effective, intermittent PrEP is likely to be attractive to users, and is also likely to be more cost-effective and less toxic than a daily regimen. In addition, sexual activity is often pre-planned for and concentrated during weekends, and is then usually not permanent, thus if this intermittent PrEP strategy is proven effective, high-risk individuals might be likely to adapt their behavior to it. Indeed, in a recent online French survey of MSM, 62.8% of 939 seronegative MSM favored ‘on-demand’ PrEP compared with only 24.6% who favored daily PrEP [23]. Interest for ‘on-demand’ PrEP was also reported in another study [33]. This strategy of event-based dosing seems best suitable for MSM who more frequently use sexual networking websites, with only 15% of them having anal sex more than 3 days a week [34]. This intermittent strategy might also be particularly attractive in young MSM because a fairly high proportion (58%) reported being intermittently adherent to PrEP [35].

Intermittent PrEP could also be designed as a fixed weekly regimen. This would have the advantage of not being related to sexual activity, and therefore would be potentially less prone to missed doses in cases when sexual activity could not be anticipated. Indeed, TDF and FTC both have long intracellular half-lives, suggesting that less than daily dosing could be sufficient to provide similar protection to a daily regimen. Interestingly, when comparing TFV-DP concentrations in the PBMCs of participants in the active arm of IPrEx who remained uninfected with those obtained in healthy volunteers receiving different TDF/FTC dosing regimens, Anderson *et al*. suggested that TDF/FTC regimens with at least four tablets/week would achieve TFV-DP concentrations in PBMCs, associated with a 90% reduction in the risk of HIV acquisition in IPrEx [36]. Even those receiving only two tablets/week could get some degree of protection against HIV infection. Trials are under way to assess the pharmacokinetics and adherence rate to these fixed-dose intermittent regimens [31].

Other modes of PrEP delivery could also be attractive for intermittent use. In particular, parenteral injections of long-acting antiretroviral agents could be a way to overcome the issue of PrEP adherence [37]. Preliminary studies in monkeys have shown the benefit of this strategy using monthly intramuscular injections [38]. Finally, other modes of PrEP delivery for men (rectal gels with TDF) and women (antiretroviral-containing gels, films, or rings) are being assessed, but are beyond the scope of this review.

However, the efficacy assessment of these new PrEP regimens will raise funding, logistical, and ethical issues. Prevention studies are complex studies to perform, need strong community engagement to enroll large numbers of participants, and should offer to all participants the best standard of prevention.

## Conclusions

Oral PrEP with anti-retroviral drugs is a new biomedical tool that could help reduce the risk of HIV infection in high-risk individuals. Because of the challenges and limitations of the current daily PrEP regimens with TDF/FTC combinations, this strategy has not yet been implemented as a public-health strategy to reduce the continuing high number of new HIV infections. More research and new PrEP strategies have to be assessed [39]. In this regard, the outcome of current ongoing trials in Europe and the USA with oral PrEP, demonstration projects in the USA, and open-label extension of already completed placebo-controlled trials will be essential. In addition, PrEP should not be seen as an alternative to current HIV preventive strategies but rather as a complementary tool that people might want to use to further reduce their risk of HIV acquisition. PrEP should therefore be delivered within appropriate settings, where other prevention measures could also be reinforced and regular testing for HIV infection and monitoring of PrEP safety is available.

## Abbreviations

ART: Antiretroviral therapy; FDA: Food and Drug Administration; FTC: Emtricitabine; HIV: Human immunodeficiency virus; IPERGAY: Intervention Préventive de l’Exposition aux Risques avec et pour les Gays; iPrEx: Chemoprophylaxis for HIV Prevention in Men who have Sex with Men; iPrEx-OLE: IPrEX Open Label Extension; MSM: Men who have sex with men; QALY: Quality-adjusted life-year; PBMC: Peripheral blood mononuclear cell; PrEP: Pre-exposure prophylaxis; PROUD: PRe-exposure Option for reducing HIV in the UK: an open-label randomisation to immediate or Deferred daily Truvada for HIV negative gay men; STI: Sexually transmitted infection; TDF: Tenofovir disoproxil fumarate; VOICE: Vaginal and Oral Interventions to Control the Epidemic.

## Competing interests

JMM is the principal investigator of the ANRS Ipergay PrEP trial in MSM conducted in France and Canada, and has received honoraria from Gilead and Merck for advisory boards on Prevention. All other authors are also involved in the ANRS Ipergay trial.

## Authors’ contributions

All authors contributed to the manuscript. JMM wrote the first draft and all other authors amended the manuscript. All authors read and approved the final manuscript.

## Authors’ information

This paper was presented in part at the XIth international congress on HIV and Drug Therapy in HIV Infection, Glasgow, November 11–15, 2012.

## Note

This article is part of the cross journal collection *HIV 30 years on*. Other articles in this series can be found at http://www.biomedcentral.com/series/HIV_30.
